# The association between myopia and health-related quality of life among Chinese children in primary and secondary school: A cross-sectional study

**DOI:** 10.1371/journal.pone.0324123

**Published:** 2025-05-27

**Authors:** Shijie Yu, Wei Sun, Hongpo Yin, Jianfeng Wu, Hongsheng Bi

**Affiliations:** 1 Shandong University of Traditional Chinese Medicine, Medical College of Optometry and Ophthalmology, Jinan, China; 2 Affiliated Eye Hospital of Shandong University of Traditional Chinese Medicine, Jinan, China; 3 Shandong Provincial Key Laboratory of Integrated Traditional Chinese and Western Medicine for Prevention and Therapy of Ocular Diseases, Shandong Provincial Clinical Medical Research Center of Optometry and Adolescent Low Vision Prevention and Control, Shandong Engineering Technology Research Center of Visual Intelligence, Jinan, China; University of Huddersfield, UNITED KINGDOM OF GREAT BRITAIN AND NORTHERN IRELAND

## Abstract

**Background:**

Previous study on the relationship between myopia and health-related quality of life (HRQOL) among children was only conducted within hospital setting, and this relationship in school environment remained unknown. This study aimed to investigate the association between myopia and HRQOL among Chinese children aged 6–15 years in primary and secondary school.

**Methods:**

This cross-sectional study included 1,634 children, all of whom underwent routine eye examinations including cycloplegic autorefraction. The EQ-5D-Y was used to assess HRQOL. Multiple linear regression models were performed to investigate the association of myopia with EQ-5D-Y utility index (UI) values and visual analogue scale (VAS) scores.

**Results:**

Among all children, 695 (43.53%) were diagnosed with myopia ranging from -0.5 to -10.5 diopters; the mean age was 9.38 ± 2.23 years old; 838 (51.29%) were boys, and 796 (48.71%) were girls. Compared with emmetropic children, myopic children had a smaller proportion of problems with self-care and a larger proportion of problems with pain/discomfort and anxiety/depression. Children with myopia had significantly lower UI values [β = -0.008, 95% confidence interval (CI): -0.016, 0.000] and VAS scores (β = -1.300, 95%CI: -2.522, -0.078) compared to their emmetropic peers. The self-evaluation of eye health was positively associated with both UI values and VAS scores. Furthermore, decreases in UI values and VAS scores were associated to the onset of myopia, and were more pronounced in children with myopia progression.

**Conclusions:**

This study found a significant association between myopia and worse HRQOL in primary and secondary school children. These findings highlight that governments and society should pay attention to the HRQOL of myopic children.

## Introduction

As the prevalence of myopia has increased dramatically worldwide, so has the associated health burden [1]. It is projected that by 2050, myopia would affect half of the global population (around 5 billion) [2]. Globally myopia incurs direct healthcare costs and productivity losses, which are estimated to be in the billions of dollars [3]. Grades 1–7 (age from 6–13 years) are critical periods characterized by a rapid rise in the prevalence of myopia among school children [4]. In Shandong, China, the prevalence of myopia among children and adolescents is reported to be 58.66% [5]. Myopia can cause blurred vision especially at distance, interfering with daily activities and subsequent symptoms such as frustration, depression and even sleep disorders, reducing health-related quality of life (HRQOL) [6,7]. Refractive errors, predominantly myopia, account for one-fifth of all causes of blindness worldwide [8]. The number of disability adjusted life years (DALYs) due to uncorrected refractive error among adolescents globally has increased by 8% from 814,261.98 in 1990–879,736.05 in 2019 [9]. Increased burden of disease is usually associated with decreased HRQOL [10,11]. Therefore, it is valuable to explore the association between myopia and HRQOL among children. Han and co-workers found that children with high myopia had significantly lower HRQOL [12]. In China, due to economic factors, some families choose optical stores as places for children’s visual acuity correction instead of hospitals, and hospital-based study designs may result in selection bias. As children spend most of their time in school, it is logical to explore this association within a school environment.

Developed by EuroQol Group, the EuroQol five-dimension questionnaire for youth (EQ-5D-Y) is a child-friendly version of the tool for measuring HRQOL in children aged 4–15 years [13]. Application of the EQ-5D-Y in a multinational sample of children and adolescents have shown great feasibility, reliability, and validity [14]. Due to its simplicity, great measurement performance and ability to capture a wide range of health conditions, EQ-5D-Y is frequently used in different pediatric population samples [15–17]. Azuara-Blanco and co-workers used EQ-5D-Y in myopic children aged 6–12 years to detect changes in their HRQOL in a randomized controlled trial of low-dose (0.01%) of atropine eye-drops to reduce myopia progression [18]. More so, EQ-5D-Y is capable of generating utility index (UI) values, thereby quantifying HRQOL effectively [19]. EQ-5D-Y has been employed in a variety of settings, including clinical trials, everyday clinical settings, population studies, and health economic evaluations [13,20–22]. In summary, the EQ-5D-Y serves as a versatile instrument, offering valuable insights into the health conditions, in particular HRQOL of children, thereby can be used to inform healthcare decisions and strategies.

This cross-sectional study tried to understand the current status of HRQOL in myopic children and to examine the association between myopia and HRQOL among children in primary and secondary school aged 6–15 years by using EQ-5D-Y as part of an ophthalmological epidemiological survey.

## Materials and Methods

### Sampling and settings

This cross-sectional study was conducted from September to October in 2023 as part of an ophthalmological epidemiological survey investigating myopia prevention and control in Huantai County, Zibo City, Shandong Province, China. The data collection of this was done via wenjuanxing platform (https://www.wjx.cn/app/survey.aspx). Two schools were randomly selected from the 14 nine-year compulsory schools in this area. Firstly, children underwent detailed eye examinations at school. Then, children were provided with detailed instructions for the questionnaire, and then scanned the QR code on their smartphones to complete the questionnaire under parental supervision after school. The inclusion criteria were as follows: both children and their parents consented to participate in the eye examinations and questionnaire survey, and children were not suffering from any serious physical or mental illness. The exclusion criteria were as follows: children who either did not complete the routine eye examinations or were diagnosed with a significant medical condition such as body disorders and cancers; children diagnosed with hyperopia, and children who either did not complete the questionnaire or whose questionnaires with inconsistent or confusing answers were deemed abnormal.

**Fig 1:**
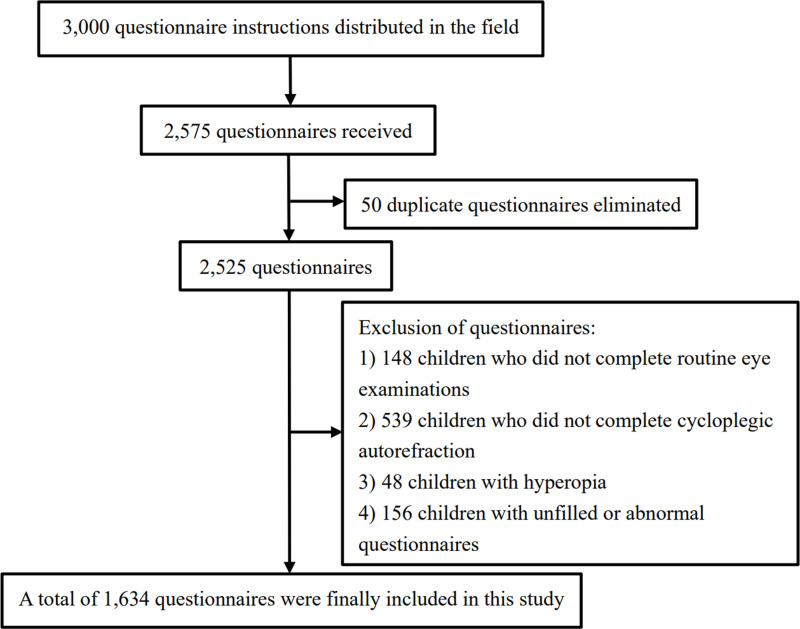
Flow diagram of questionnaire distribution and collation.

In total, 3,000 questionnaires were distributed, and 2,575 questionnaires were returned. Initially, we excluded 50 duplicate questionnaires. From those, 148 children who did not complete routine eye examinations, 539 children who did not complete cycloplegic autorefraction, 48 children with hyperopia, and 156 children with unfilled or abnormal questionnaires were also excluded. Consequently, a total of 1,634 questionnaires were deemed valid and included in the final analysis of this study (see Fig 1).

### Measurement

#### Eye examinations and visual acuity surveys.

Firstly, all children were subjected to a slit lamp examination and funduscopic examination performed by two experienced ophthalmologists. Following this, a series of detailed visual acuity tests were administered by skilled optometrists. These tests encompassed uncorrected visual acuity tests at a standard testing distance of 4 meters, using the ETDRS Log MAR E vision chart (Precision Vision, Villa Park, Illinois, USA), non-contact tonometry (Topcon CT80; Topcon Corp., Tokyo, Japan), and autorefraction both with and without cycloplegia (Nidek ARK-1, CO., LTD, Japan). The cycloplegic eye drops used in this study were cyclopentolate hydrochloride ophthalmic solution (1%) (Alcon, Fort Worth, TX, USA) [23]. The administration protocol involved dispensing one drop every five minutes, for a total of three doses [23]. After the administration, the children were instructed to rest with their eyes closed for a period of 30 minutes. Observations were subsequently made regarding the pupillary light reflex and diameter. In cases where the pupillary reflex was absent or the pupil diameter was greater than 6 mm, an autorefraction test was conducted. If the pupil diameter was less than 6 mm, additional eye drops were administered. After a waiting period of 10 minutes, the autorefraction test was performed.

The Spherical equivalence (SE) is calculated by adding the spherical refractive error to half of the cylindrical refractive error. Myopia is defined as an SE of ≤ -0.50 D post-cycloplegia, while emmetropia is defined as an SE that is > **−**0.50 D and <+2.00 D post-cycloplegia [24,25]. In addition, the children were asked about eye health self-evaluation and visual acuity change in previous year through two questions in the questionnaire. The first question was, “What is your opinion on your eye health?” with response options being “bad”, “general”, and “good”. Younger children needed to understand this question and express their opinions required parental guidance. The second question was, “which one is likely to be your visual acuity change in previous year?” with response options being “no change”, “myopia onset”, and “myopia progression”. Change in visual acuity in previous year was based on children’s perceptual feedback, and answered with parental assistance.

#### Health-related quality of life.

HRQOL is measured by the EQ-5D-Y instrument, which includes five health dimensions (i.e., mobility, self-care, usual activities, pain/discomfort, and anxiety/depression). Each dimension is categorized into three levels of severity: no problems, some problems, and a lot of problems. The UI value for the EQ-5D-Y was calculated by a value set suitable for Chinese children, based on health conditions in the five dimensions [19]. It ranges from −0.089 to 1.0, with higher UI values representing higher HRQOL based on results reported by Yang and colleagues [19]. Additionally, the EuroQol visual analogue scale (EQ-VAS) score was used, which provides a quantitative assessment of children’s self-rated health, on a scale from 0 (representing the worst health condition imaginable) to 100 (representing the best health condition imaginable) [13].

#### Covariates.

Covariates were constituted with children’s characteristics and family’s characteristics. The children’s characteristics included gender (boy/girl), age (years), nation (Han/others), type of medical insurance (basic medical insurance for urban and rural residents/commercial insurance/others), presence of underlying health condition (no/yes), school bullying (no/yes), frequency of exposure to second-hand smoke (few/sometimes), number of friends (<3/3–5/ > 5), academic performance (below average/average/above average), exercise time per week (<1 hour/1–5 hours/5–10 hours/ > 10 hours), evaluation of dietary status (unhealthy/ healthy/very healthy), and extracurricular reading time per day (<1 hour/1–2 hours/ > 2 hours). The family’s characteristics included average age of parents (years), parental education level (primary or secondary school/high or vocational high school/junior college, university or above), relationship between children and their parents (bad/ general/good), total income per year (Chinese Yuan) (<30,000/30,000–60,000/60,000–90,000/90,000–120,000/ > 120,000), and parents working in other cities (0/1/2). It is noted that the covariates were answered by a parent living with child according to real situation.

### Statistical analyses

Descriptive analyzes were performed to summarize the characteristics of study participants. The participants were divided into two groups based on the refractive status: emmetropia or myopia. Continuous variables were shown as mean plus/minus standard deviation, and categorical variables were shown as numbers (percentages). Group comparisons were conducted using the Kruskal-Wallis test for continuous variables and the Chi-Square test for categorical variables. Multiple linear regression analysis models were used to investigate the associations between the characteristics of myopia and HRQOL (both UI and VAS). We initially started with an unadjusted model. Subsequently, we adjusted for a series of covariates on children and family.

To strengthen the credibility of our findings, the refractive status of children who underwent routine eye examinations, but did not participate in cycloplegic autorefraction was assessed. Data from non-cycloplegic autorefraction was utilized for this assessment. Following this, statistical analyses were conducted on the entire children population again. The results of these analyses are presented in the supplementary material (see S1 File).

All statistical analyses were performed using the SPSS software (IBM Corp, Version 27.0, United States of America). The Akaike’s information criterion (AIC) was utilized to assess the fit of the model. A p-value less than 0.05 in a two-sided test was considered indicative of statistical significance.

### Ethics statement

This study, which involved human participants, underwent a review and received approval from the Ethics Committee of the Affiliated Eye Hospital of Shandong University of Traditional Chinese Medicine (HEC-KS-202305KY). The child participants were introduced to an informed assent before the questionnaires were distributed, and the assenting participants were given the questionnaires. The parents or next of kin of the participants provided written informed consent for their participation in this study.

## Results

Table 1 shows the characteristics of all children (n = 1,634), along with the comparative analysis between the emmetropic (n = 939) and myopic (n = 695) groups. The mean age of all children was 9.38 ± 2.23 years, with boys constituting 51.29% of the sample. The mean SE was + 0.66 ± 0.56 diopters in the group of emmetropia, and -2.28 ± 1.54 diopters in the group of myopia. The mean ETDRS score was 80.13 ± 6.60 in the group of emmetropia, and 69.05 ± 11.35 in the group of myopia. In terms of eye health self-evaluation, in the group of emmetropia, 10 (1.06%) children reported bad eye health self-evaluation, 121 (12.89%) children reported general eye health self-evaluation, and 808 (86.05%) children reported good eye health self-evaluation. In the group of myopia, 133 (19.14%) children reported bad eye health self-evaluation, 332 (47.77%) children reported general eye health self-evaluation, and 230 (33.09%) children reported good eye health self-evaluation. Among all children, 1,168 (71.48%) children reported no change in visual acuity in previous year, 345 (21.11%) children reported myopia onset in visual acuity in previous year, and 121 (7.41%) children reported myopia progression in visual acuity in previous year.

**Table 1:** Characteristics of children in the study.

	Total (1,634)	Emmetropia (939)	Myopia (695)	*P*-value^*^
**Children’s characteristics**				
**Gender**				0.081
Boy	838 (51.29%)	499 (53.14%)	339 (48.78%)	
Girl	796 (48.71%)	440 (46.86%)	356 (51.22%)	
**Age, years (sd)**	9.38 (2.23)	8.42 (1.94)	10.67 (1.92)	**<0.001**
**Nation**				0.315
Han	1,626 (99.51%)	933 (99.36%)	693 (99.71%)	
Others	8 (0.49%)	6 (0.64%)	2 (0.29%)	
**Type of medical insurance**				0.058
Basic medical insurance for urban and rural residents	1,413 (86.47%)	814 (86.69%)	599 (86.19%)	
Commercial insurance	171 (10.47%)	104 (11.08%)	67 (9.64%)	
Others	50 (3.06%)	21 (2.24%)	29 (4.17%)	
**SE, diopters (sd)**	−0.59 (1.82)	0.66 (0.56)	−2.28 (1.54)	**<0.001**
**ETDRS (sd)**	75.42 (10.48)	80.13 (6.60)	69.05 (11.35)	**<0.001**
**Eye health self-evaluation**				**<0.001**
Bad	143 (8.75%)	10 (1.06%)	133 (19.14%)	
General	453 (27.72%)	121 (12.89%)	332 (47.77%)	
Good	1,038 (63.53%)	808 (86.05%)	230 (33.09%)	
**Visual acuity change in previous year** ^**^				
No change	1,168 (71.48%)			
Myopia onset	345 (21.11%)			
Myopia progression	121 (7.41%)			
**Presence of underlying health condition**				0.454
No	1,627 (99.57%)	934 (99.47%)	693 (99.71%)	
Yes	7 (0.43%)	5 (0.53%)	2 (0.29%)	
**School bullying**				0.931
No	1,610 (98.53%)	925 (98.51%)	685 (98.56%)	
Yes	24 (1.47%)	14 (1.49%)	10 (1.44%)	
**Frequency of exposure to second-hand smoke**				**0.015**
Few	1,198 (73.32%)	667 (71.03%)	531 (76.40%)	
Sometimes	436 (26.68%)	272 (28.97%)	164 (23.60%)	
**Number of friends**				0.063
<3	116 (7.10%)	69 (7.35%)	47 (6.76%)	
3–5	689 (42.17%)	417 (44.41%)	272 (39.14%)	
>5	829 (50.73%)	453 (48.24%)	376 (54.10%)	
**Academic performance**				**<0.001**
Below average	47 (2.88%)	21 (2.24%)	26 (3.74%)	
Average	432 (26.44%)	214 (22.79%)	218 (31.37%)	
Above average	1,155 (70.69%)	704 (74.97%)	451 (64.89%)	
**Exercise time per week**				0.213
<1 hour	337 (20.62%)	183 (19.49%)	154 (22.16%)	
1-5 hours	748 (45.78%)	425 (45.26%)	323 (46.47%)	
5-10 hours	355 (21.73%)	208 (22.15%)	147 (21.15%)	
>10 hours	194 (11.87%)	123 (13.10%)	71 (10.22%)	
**Evaluation of dietary status**				0.559
Unhealthy	19 (1.16%)	10 (1.06%)	9 (1.29%)	
Healthy	211 (12.91%)	128 (13.63%)	83 (11.94%)	
Very healthy	1,404 (85.92%)	801 (85.30%)	603 (86.76%)	
**Extracurricular reading time per day**				**<0.001**
<1 hour	938 (57.41%)	578 (61.55%)	360 (51.80%)	
1-2 hours	588 (35.99%)	313 (33.33%)	275 (39.57%)	
>2 hours	108 (6.61%)	48 (5.11%)	60 (8.63%)	
**Family’s characteristics**				
**Average age of parents, years (sd)**	38.46 (5.07)	37.95 (4.93)	39.15 (5.18)	**<0.001**
**Parental education level**				**0.032**
Primary or secondary school	574 (35.13%)	305 (32.48%)	269 (38.71%)	
High or vocational high school	623 (38.13%)	375 (39.94%)	248 (35.68%)	
Junior college/university or above	437 (26.74%)	259 (27.58%)	178 (25.61%)	
**Relationship between children and their parents**				0.307
Bad	14 (0.86%)	9 (0.96%)	5 (0.72%)	
General	212 (12.97%)	112 (11.93%)	100 (14.39%)	
Good	1,408 (86.17%)	818 (87.11%)	590 (84.89%)	
**Total income per year, Chinese Yuan**				0.149
<30,000	249 (15.24%)	144 (15.34%)	105 (15.11%)	
30,000-60,000	474 (29.01%)	273 (29.07%)	201 (28.92%)	
60,000-90,000	340 (20.81%)	189 (20.13%)	151 (21.73%)	
90,000-120,000	301 (18.42%)	161 (17.15%)	140 (20.14%)	
>120,000	270 (16.52%)	172 (18.32%)	98 (14.10%)	
**Parents working in other cities**				0.384
0	1,401 (85.74%)	802 (85.41%)	599 (86.19%)	
1	189 (11.57%)	115 (12.25%)	74 (10.65%)	
2	44 (2.69%)	22 (2.34%)	22 (3.17%)	

* continues variables were compared by Kruskal–Wallis test; categorical variables were compared by Chi-Square test

** adoption by self-reporting

Table 1 shows that significant differences were observed in terms of age, SE, ETDRS, eye health self-evaluation, frequency of exposure to second-hand smoke, academic performance, extracurricular reading time per day, average age of parents, parental education level among emmetropic group and myopic group. Among the myopic children, the proportion of children with an older age, lower ETDRS scores, lower SE, worse eye health self-evaluation, fewer frequency of exposure to second-hand smoke, better academic performance, more extracurricular reading time per day, older average age of parents, lower parental education level was higher than that among emmetropic children. No significant differences were found between two groups in other characteristics.

Table 2 shows the characteristics of EQ-5D-Y of all children and the comparison between emmetropia and myopia. There were significant differences in self-care, pain/discomfort, anxiety/depression, UI, and VAS between the two groups. Among myopic children, the proportion of children that reported fewer problems in looking after myself, more problems in having pain or discomfort, and feeling worried, sad or unhappy was significantly higher than that among emmetropic children. The mean UI values were 0.96 ± 0.07, 0.97 ± 0.07, and 0.95 ± 0.08 for all children, emmetropic children, and myopic children, respectively. The corresponding mean VAS scores were 94.14 ± 11.10, 94.84 ± 10.79, and 93.21 ± 11.45, respectively. The myopic group exhibited significantly higher UI values (*P* = 0.002) and VAS scores (*P* < 0.001) compared to the emmetropic group. No significant between-group differences were observed in other characteristics of EQ-5D-Y.

**Table 2:** Characteristics of EQ-5D-Y of children in the study.

EQ-5D-Y	Total (1,634)	Emmetropia (939)	Myopia (695)	*P*-value^*^
**Mobility (walking about)**				0.536
No problems	1,611 (98.59%)	927 (98.72%)	684 (98.42%)	
Some problems	22 (1.35%)	11 (1.17%)	11 (1.58%)	
A lot of problems	1 (0.06%)	1 (0.11%)	0 (0.00%)	
**Looking after myself**				**0.010**
No problems	1,551 (94.92%)	878 (93.50%)	673 (96.83%)	
Some problems	80 (4.90%)	59 (6.28%)	21 (3.02%)	
A lot of problems	3 (0.18%)	2 (0.21%)	1 (0.14%)	
**Doing usual activities**				0.775
No problems	1,521 (93.08%)	877 (93.40%)	644 (92.66%)	
Some problems	110 (6.73%)	60 (6.39%)	50 (7.19%)	
A lot of problems	3 (0.18%)	2 (0.21%)	1 (0.14%)	
**Having pain or discomfort**				**0.003**
No problems	1,430 (87.52%)	843 (89.78%)	587 (84.46%)	
Some problems	200 (12.24%)	93 (9.90%)	107 (15.40%)	
A lot of problems	4 (0.24%)	3 (0.32%)	1 (0.14%)	
**Feeling worried, sad or unhappy**				**0.003**
No problems	1,235 (75.58%)	737 (78.49%)	498 (71.65%)	
Some problems	374 (22.89%)	192 (20.45%)	182 (26.19%)	
A lot of problems	25 (1.53%)	10 (1.06%)	15 (2.16%)	
**EQ-5D-Y UI (sd)**	0.96 (0.07)	0.97 (0.07)	0.95 (0.08)	**0.002**
**EQ-5D-Y VAS (sd)**	94.14 (11.10)	94.84 (10.79)	93.21 (11.45)	**<0.001**

* continues variables were compared by Kruskal–Wallis test; categorical variables were compared by Chi-Square test.

The association between the characteristics of myopia and UI modeled by multiple linear regressions is shown in Table 3. After adjusting for covariates, it was observed that children with myopia had significantly lower UI values compared to their emmetropic counterparts (β = −0.008, 95% confidence interval [CI]: −0.016, 0.000; *P* = 0.049). As the SE increased, the UI values increased among children (β = 0.002, 95%CI: 0.000, 0.005; *P* = 0.039). Children with good eye health self-evaluation had higher UI values compared to those with general eye health self-evaluation (β = 0.014, 95%CI: 0.005, 0.022; *P* = 0.001). Children who reported myopia onset in previous year had lower UI values (β = −0.013, 95%CI: −0.022, −0.003; *P* = 0.007), and children who reported myopia progression in previous year had even lower UI values (β = −0.026, 95%CI: −0.041, −0.012; *P* < 0.001), compared to those with no change in visual acuity in previous year.

**Table 3:** Association between characteristics of myopia and UI of children in the study.

Characteristics of myopia	Unadjusted model		Adjusted model^*^	
	β (95%CI)	*P*-value	β (95%CI)	*P*-value
**Refraction status**				
Emmetropia	ref	ref	ref	ref
Myopia	−**0.011 (**−**0.018,** −**0.003)**	**0.004**	−**0.008 (**−**0.016, 0.000)**	**0.049**
**SE, diopters**	**0.003 (0.001, 0.005)**	**0.002**	**0.002 (0.000, 0.005)**	**0.039**
**ETDRS**	0.000 (0.000, 0.001)	0.238	0.000 (0.000, 0.001)	0.355
**Eye health self-evaluation**				
General	ref	ref	ref	ref
Bad	−0.012 (−0.027, 0.003)	0.124	−0.009 (−0.025, 0.006)	0.244
Good	**0.018 (0.010, 0.026)**	**<0.001**	**0.014 (0.005, 0.022)**	**0.001**
**Visual acuity change in previous year** ^**^				
No change	ref	ref	ref	ref
Myopia onset	−**0.014 (**−**0.023,** −**0.005)**	**0.001**	−**0.013 (**−**0.022,** −**0.003)**	**0.007**
Myopia progression	−**0.030 (**−**0.043,** −**0.016)**	**<0.001**	−**0.026 (**−**0.041,** −**0.012)**	**<0.001**

* adjusting for gender, age, nation, type of medical insurance, presence of underlying health condition, school bullying, frequency of exposure to second-hand smoke, number of friends, academic performance, evaluation of dietary status, exercise time per week, extracurricular reading time per day, average age of parents, parental education level, relationship between children and their parents, total income per year, parents working in other cities

** adoption by self-reporting

The association between the characteristics of myopia and VAS modeled by multiple linear regressions is shown in Table 4. After adjusting for covariates, it was observed that myopic children had significantly lower VAS scores compared to their emmetropic counterparts (β = −1.300, 95%CI: −2.522, −0.078; *P* = 0.037). As the SE increased, the VAS scores increased among children (β = 0.424, 95%CI: 0.081, 0.767; *P* = 0.015). Similarly, the VAS scores increased with the ETDRS scores increasing among children (β = 0.100, 95%CI: 0.049, 0.151; *P* < 0.001). Children with bad eye health self-evaluation had lower VAS scores (β = −2.615, 95%CI: −5.050, −0.179; *P* = 0.035), and children with good eye health self-evaluation had higher VAS scores (β = 1.971, 95%CI: 0.782, 3.160; *P* = 0.001), compared to those with general eye health self-evaluation. Children who reported myopia progression in previous year had lower VAS scores (β = −2.694, 95%CI: −4.883, −0.505; *P* = 0.016), compared to those with no change in visual acuity in previous year.

**Table 4:** Association between characteristics of myopia and VAS of children in the study.

Characteristics of myopia	Unadjusted model		Adjusted model^*^	
	β (95%CI)	*P*-value	β (95%CI)	*P*-value
**Refraction status**				
Emmetropia	ref	ref	ref	ref
Myopia	−**1.626 (**−**2.713,** −**0.539)**	**0.003**	−**1.300 (**−**2.522,** −**0.078)**	**0.037**
**SE, diopters**	**0.521 (0.226, 0.816)**	**0.001**	**0.424 (0.081, 0.767)**	**0.015**
**ETDRS**	**0.111 (0.060, 0.162)**	**<0.001**	**0.100 (0.049, 0.151)**	**<0.001**
**Eye health self**−**evaluation**				
General	ref	ref	ref	ref
Bad	**−3.540 (−5.936, −1.145)**	**0.004**	**−2.615 (−5.050, −0.179)**	**0.035**
Good	**2.606 (1.441, 3.770)**	**<0.001**	**1.971 (0.782, 3.160)**	**0.001**
**Visual acuity change in previous year** ^**^				
No change	ref	ref	ref	ref
Myopia onset	−1.027 (−2.344, 0.290)	0.126	−0.827 (−2.192, 0.538)	0.235
Myopia progression	**−3.550 (−5.620, −1.480)**	**0.001**	**−2.694 (−4.883, −0.505)**	**0.016**

* adjusting for gender, age, nation, type of medical insurance, presence of underlying health condition, school bullying, frequency of exposure to second-hand smoke, number of friends, academic performance, evaluation of dietary status, exercise time per week, extracurricular reading time per day, average age of parents, parental education level, relationship between children and their parents, total income per year, parents working in other cities

** adoption by self-reporting

## Discussion

To the best of our knowledge, this is the first school-based study investigating the association between myopia and HRQOL among Chinese children in school setting. The primary and secondary school periods are critical for the onset and progression of myopia, making it imperative to investigate the association between myopia and HRQOL during these periods. These findings revealed a negative association between myopia and HRQOL. When compared to children with general eye health self-evaluation, those with bad eye health self-evaluation generally exhibited lower HRQOL. On the other hand, children with good eye health self-evaluation generally exhibited higher HRQOL. In contrast to those without changes in visual acuity, children with myopia onset had lower HRQOL, and this was further lower in children with progressive myopia. Myopic children’s reduced health conditions bring many inconveniences to their daily activities. It was exhibited by declines in multiple dimensions of EQ-5D-Y, resulting in lower HRQOL scores. Hence, regular detection and timely intervention for myopia among children, especially from an early age, are critical.

Significant differences in several dimensions of EQ-5D-Y were observed between the myopic and emmetropic groups. Notably, a smaller proportion of myopic children reported problems in self-care compared to emmetropic children. This could be attributed to the fact that myopic blurred vision affects children’s ability to take care of themselves [26]. Furthermore, a larger proportion of myopic children reported problems with pain/discomfort and anxiety/depression compared to emmetropes. Similar to our study, Thorud and colleagues found a significant association between uncorrected low myopia and reduced academic performance and increased musculoskeletal pain among school children [27]. Also, Huang and colleagues found that myopic students scored 0.12 standard deviations higher in the Center for Epidemiologic Studies Depression (CES-D) scale compared to non-myopic students, suggesting a higher likelihood of depression among older students with myopia [6]. Hence, ocular discomfort can have an impact in mental well-being. It is crucial to focus on eye health as well as mental health.

This study found that myopia was associated with worse HRQOL among children in primary and secondary school. As one of the most common chronic eye conditions in childhood, myopia can lead to blurred vision, thereby disrupting daily activities [28,29]. A hospital-based study conducted by Han and colleagues found that the total Pediatric Quality of Life Inventory (PedsQL) scores of children with high myopia were significantly lower than those with low myopia; as the SE increased, the total PedsQL scores were significantly higher among children [12]. Since this study was conducted in a school setting, and lacked a sufficient sample size for high myopia, we limited our comparison to the myopic and emmetropic groups. Our results indicated that children with myopia had significantly lower UI values and VAS scores, and as the SE and ETDRS scores increased, children tended to have higher UI values and VAS scores. Given that childhood is a critical period of growth, implementing interventions, such as social and cultural engagement, is critical in order to improve overall quality of life.

The eye health self-evaluation in children was aligned with their HRQOL. It was noteworthy that myopic children had significantly worse eye health self-evaluation compared to their emmetropic counterparts. This difference could be attributed to physical discomfort, visual limitations, and psychosocial challenges associated with myopia, such as difficulties in participation in sports, increased dependence on corrective lenses, and potential stigma [3]. Children with good eyes health self-evaluation generally had a higher HRQOL, while those with bad eye health self-evaluation generally had a lower HRQOL. This is a key finding that highlights the significance of perceived eye health and its association with the overall well-being of children. Thus, greater emphasis should be placed on eye health education for children in primary and secondary schools.

The decline in visual acuity was found to have a negative association with HRQOL among children. It is well established that optimal visual acuity is vital for children [30]. Children reporting declines in visual acuity might encounter learning obstacles if they were unable to see clearly in class [31]. Our study indicated that nearly one-third of all children reported the onset or progression of myopia in the past year. Generally, children who reported a decline in visual acuity, especially those with myopia progression, had a lower HRQOL. Parents should be advised to schedule regular visual acuity check-ups for their children, and ensure timely treatment of any vision-related issues, which can positively impact children’s HRQOL [12].

As additional analyses for this study, data obtained from non-cycloplegic autorefraction eye examinations performed on children was incorporated. The results were consistent with our observations derived from children who underwent cycloplegic autorefraction (see S1 File). When compared to other studies on chronic diseases utilizing the EQ-5D-Y scale, the decrease in HRQOL scores for myopic children was less marked. For instance, Sun and colleagues reported a mean utility index (UI) value of 0.88 and a mean visual analogue scale (VAS) score of 85.8 among Chinese pediatric patients with hematological malignancies [32]. Similarly, López-Bastida and colleagues found a mean UI value of 0.95 and a mean VAS score of 86.1 among pediatric patients with type 1 diabetes mellitus [33]. Myopia is less life-threatening and does not necessitate adherence to medication. Therefore, children with myopia do not report a significant reduction in HRQOL compared to those with severe chronic diseases.

This study provides valuable epidemiological evidence for the association between myopia and HRQOL among children in primary and secondary school. In the process of implementing strategies on myopia prevention and control, it is important to consider the overall health status of children. For those affected by myopia, the provision of essential medical support is necessary. There are several limitations in this study. First, this study was conducted within a single region, which inherently restricted the generalizability of our findings. Nonetheless, our conclusions can serve as a valuable reference for regions with a high prevalence of myopia [28]. Second, face-to-face surveys are difficult due to the fact that some questions, such as parents’ age and family income, are difficult for young children to answer. Parents were allowed to help their children complete the questionnaire together at home. Consequently, it inevitably leads to recall bias such as family income and exercise time, and affects the reality of data such as the relationship between children and parents. Third, some children were unable to participate in cycloplegic autorefraction due to various reasons such as schooling and competitions. To mitigate this, additional analyses using data from non-cycloplegic autorefraction was conducted, and the results supported the initial findings. Fourth, this study employed a cross-sectional design, which makes it challenging to establish a causal relationship between myopia and the decline in HRQOL. Future research employing longitudinal designs may help to further elucidate this relationship.

## Conclusions

In summary, this research underscores that myopia is closely associated with reduced HRQOL, particularly in children reporting perceived myopia progression. The importance of children’s perceived self-evaluation of eye health is emphasized. Comprehensive public health strategies should be advocated for formulation. These strategies should extend beyond myopia prevention and control, taking into account the overall health status of children, and integrating HRQOL evaluations into school-based myopia prevention and control. Future research employing longitudinal and larger, more diverse cohorts will be instrumental in further clarifying the causal relationship between myopia and HRQOL.

## Supporting information

S1 File The results of analyses among children with and without cycloplegic autorefraction. (DOC)
