# Supplementary material for: The association between myopia and health-related quality of life among Chinese children in primary and secondary school: A cross-sectional study
Source: PLoS One. 2025 May 27;20(5):e0324123. doi: 10.1371/journal.pone.0324123 (PMC12111671; doi:10.1371/journal.pone.0324123)
Supplement: S1 File — (DOC) [file pone.0324123.s001.docx]

3,000 questionnaire instructions distributed in the field

2,575 questionnaires received

50 duplicate questionnaires eliminated

2,525 questionnaires

Exclusion of questionnaires:

1) 148 children who did not complete routine eye examinations

2) 51 children with hyperopia

3) 175 children with unfilled or abnormal questionnaires

A total of 2,151 questionnaires were finally included in this study

**Fig S1. Flow diagram of questionnaire distribution and collation**

**Table S1. Characteristics of** **children in the study**

|  | **Total**  **(2,151)** | **Emmetropia**  **(1,041)** | **Myopia**  **(1,110)** | ***P*-value*** |
| --- | --- | --- | --- | --- |
| **Children’s characteristics** |  |  |  |  |
| **Gender** |  |  |  | **0.011** |
| Boy | 1,082(50.30%) | 553(53.12%) | 529(47.66%) |  |
| Girl | 1,069(49.70%) | 488(46.88%) | 581(52.34%) |  |
| **Age, years (sd)** | 9.94(2.56) | 8.54(2.10) | 11.26(2.23) | **<0.001** |
| **Nation** |  |  |  | 0.462 |
| Han | 2,141(99.54%) | 1,035(99.42%) | 1,106(99.64%) |  |
| Others | 10(0.46%) | 6(0.58%) | 4(0.36%) |  |
| **Type of medical insurance** |  |  |  | 0.103 |
| Basic medical insurance for urban and rural residents | 1,855(86.24%) | 896(86.07%) | 959(86.40%) |  |
| Commercial insurance | 225(10.46%) | 118(11.34%) | 107(9.64%) |  |
| Others | 71(3.30%) | 27(2.59%) | 44(3.96%) |  |
| **SE, diopters (sd)** | -1.02(2.06) | 0.61(0.58) | -2.56(1.74) | **<0.001** |
| **ETDRS (sd)** | 75.17(11.07) | 80.13(6.61) | 70.52(12.32) | **<0.001** |
| **Eye health self-evaluation** |  |  |  | **<0.001** |
| Bad | 246(11.44%) | 11(1.06%) | 235(21.17%) |  |
| General | 623(28.96%) | 132(12.68%) | 491(44.23%) |  |
| Good | 1,282(59.60%) | 898(86.26%) | 384(34.59%) |  |
| **Visual acuity change in previous year**** |  |  |  |  |
| No change | 1,413(65.69%) |  |  |  |
| Myopia onset | 523(24.31%) |  |  |  |
| Myopia progression | 215(10.00%) |  |  |  |
| **Presence of underlying health condition** |  |  |  | 0.171 |
| No | 2,141(99.54%) | 1,034(99.33%) | 1,107(99.73%) |  |
| Yes | 10(0.46%) | 7(0.67%) | 3(0.27%) |  |
| **School bullying** |  |  |  | 0.593 |
| No | 2,117(98.42%) | 1,023(98.27%) | 1,094(98.56%) |  |
| Yes | 34(1.58%) | 18(1.73%) | 16(1.44%) |  |
| **Frequency of exposure to second-hand smoke** |  |  |  | **0.001** |
| Few | 1,603(74.52%) | 742(71.28%) | 861(77.57%) |  |
| Sometimes | 548(25.48%) | 299(28.72%) | 249(22.43%) |  |
| **Number of friends** |  |  |  | **<0.001** |
| <3 | 151(7.02%) | 79(7.59%) | 72(6.49%) |  |
| 3-5 | 868(40.35%) | 460(44.19%) | 408(36.76%) |  |
| >5 | 1,132(52.63%) | 502(48.22%) | 630(56.76%) |  |
| **Academic** **performance** |  |  |  | **<0.001** |
| Below average | 78(3.63%) | 26(2.50%) | 52(4.68%) |  |
| Average | 571(26.55%) | 243(23.34%) | 328(29.55%) |  |
| Above average | 1,502(69.83%) | 772(74.16%) | 730(65.77%) |  |
| **Exercise time per week** |  |  |  | 0.305 |
| <1 hour | 429(19.94%) | 207(19.88%) | 222(20.00%) |  |
| 1-5 hours | 983(45.70%) | 466(44.76%) | 517(46.58%) |  |
| 5-10 hours | 485(22.55%) | 231(22.19%) | 254(22.88%) |  |
| >10 hours | 254(11.81%) | 137(13.16%) | 117(10.54%) |  |
| **Evaluation of dietary status** |  |  |  | **<0.001** |
| Unhealthy | 26(1.21%) | 11(1.06%) | 15(1.35%) |  |
| Healthy | 441(20.50%) | 168(16.14%) | 273(24.59%) |  |
| Very healthy | 1,684(78.29%) | 862(82.80%) | 822(74.05%) |  |
| **E****xtracurricular reading time per day** |  |  |  | **<0.001** |
| <1 hour | 1,191(55.37%) | 641(61.58%) | 550(49.55%) |  |
| 1-2 hours | 814(37.84%) | 350(33.62%) | 464(41.80%) |  |
| >2 hours | 146(6.79%) | 50(4.80%) | 96(8.65%) |  |
| **Family’s characteristics** |  |  |  |  |
| **Average age of parents, years (sd)** | 38.90(5.25) | 38.10(5.04) | 39.64(5.34) | **<0.001** |
| **Parental education level** |  |  |  | **<0.001** |
| Primary or secondary school | 804(37.38%) | 343(32.95%) | 461(41.53%) |  |
| High or vocational high school | 799(37.15%) | 416(39.96%) | 383(34.50%) |  |
| Junior college/university or above | 548(25.48%) | 282(27.09%) | 266(23.96%) |  |
| **Relationship between children and their parents** |  |  |  | **0.032** |
| Bad | 21(0.98%) | 9(0.86%) | 12(1.08%) |  |
| General | 294(13.67%) | 122(11.72%) | 172(15.50%) |  |
| Good | 1,836(85.36%) | 910(87.42%) | 926(83.42%) |  |
| **Total income per year, Chinese Yuan** |  |  |  | 0.087 |
| <30,000 | 340(15.81%) | 163(15.66%) | 177(15.95%) |  |
| 30,000-60,000 | 609(28.31%) | 297(28.53%) | 312(28.11%) |  |
| 60,000-90,000 | 465(21.62%) | 212(20.37%) | 253(22.79%) |  |
| 90,000-120,000 | 388(18.04%) | 178(17.10%) | 210(18.92%) |  |
| >120,000 | 349(16.23%) | 191(18.35%) | 158(14.23%) |  |
| **Parents working in other cities** |  |  |  | 0.367 |
| 0 | 1,864(86.66%) | 894(85.88%) | 970(87.39%) |  |
| 1 | 238(11.06%) | 125(12.01%) | 113(10.18%) |  |
| 2 | 49(2.28%) | 22(2.11%) | 27(2.43%) |  |

* continues variables were compared by Kruskal–Wallis test; categorical variables were compared by Chi-Square test

** adoption by self-reporting

**Table S2. Characteristics of EQ-5D-Y** **of children in the study**

| **EQ-5D-Y** | **Total (2,151)** | **Emmetropia (939)** | **Myopia (695)** | ***P*-value*** |
| --- | --- | --- | --- | --- |
| **Mobility (walking about)** |  |  |  | 0.195 |
| No problems | 2,114(98.28%) | 1,027(98.66%) | 1,087(97.93%) |  |
| Some problems | 36(1.67%) | 13(1.25%) | 23(2.07%) |  |
| A lot of problems | 1(0.05%) | 1(0.10%) | 0(0.00%) |  |
| **Looking after myself** |  |  |  | **<0.001** |
| No problems | 2,055(95.54%) | 972(93.37%) | 1,083(97.57%) |  |
| Some problems | 92(4.28%) | 66(6.34%) | 26(2.34%) |  |
| A lot of problems | 4(0.19%) | 3(0.29%) | 1(0.09%) |  |
| **Doing usual activities** |  |  |  | 0.734 |
| No problems | 2,006(93.26%) | 973(93.47%) | 1,033(93.06%) |  |
| Some problems | 142(6.60%) | 66(6.34%) | 76(6.85%) |  |
| A lot of problems | 3(0.14%) | 2(0.19%) | 1(0.09%) |  |
| **Having pain or discomfort** |  |  |  | **<0.001** |
| No problems | 1,853(86.15%) | 931(89.43%) | 922(83.06%) |  |
| Some problems | 290(13.48%) | 107(10.28%) | 183(16.49%) |  |
| A lot of problems | 8(0.37%) | 3(0.29%) | 5(0.45%) |  |
| **Feeling worried, sad or unhappy** |  |  |  | **<0.001** |
| No problems | 1,607(74.71%) | 818(78.58%) | 789(71.08%) |  |
| Some problems | 507(23.57%) | 213(20.46%) | 294(26.49%) |  |
| A lot of problems | 37(1.72%) | 10(0.96%) | 27(2.43%) |  |
| **EQ-5D-Y UI (sd)** | 0.96(0.08) | 0.97(0.07) | 0.95(0.09) | **<0.001** |
| **EQ-5D-Y VAS (sd)** | 93.62(11.44) | 94.70(10.78) | 92.61(11.95) | **<0.001** |

* continues variables were compared by Kruskal–Wallis test; categorical variables were compared by Chi-Square test

**Table S3. Association between characteristics of myopia and UI of children in the study**

| **Characteristics of myopia** |  | **Unadjusted model** | |  | **Adjusted model*** | |
| --- | --- | --- | --- | --- | --- | --- |
|  |  | **β (95%CI)** | ***P*-value** |  | **β (95%CI)** | ***P*-value** |
| **Refraction status** |  |  |  |  |  |  |
| Emmetropia |  | ref | ref |  | ref | ref |
| Myopia |  | **-0.013(-0.020, -0.007)** | **<0.001** |  | -0.006(-0.014, 0.001) | 0.100 |
| **SE, diopters** |  | **0.004(0.002, 0.005)** | **<0.001** |  | **0.002(0.000, 0.004)** | **0.015** |
| **ETDRS** |  | 0.000(0.000, 0.001) | 0.100 |  | 0.000(0.000, 0.000) | 0.479 |
| **Eye health self-evaluation** |  |  |  |  |  |  |
| General |  | ref | ref |  | ref | ref |
| Bad |  | **-0.027(-0.041, -0.014)** | **<0.001** |  | **-0.021(-0.034, -0.008)** | **0.001** |
| Good |  | **0.017(0.010, 0.024)** | **<0.001** |  | **0.011(0.004, 0.019)** | **0.002** |
| **Visual acuity change in previous year**** |  |  |  |  |  |  |
| No change |  | ref | ref |  | ref | ref |
| Myopia onset |  | **-0.017(-0.024, -0.009)** | **<0.001** |  | **-0.014(-0.022, -0.006)** | **0.001** |
| Myopia progression |  | **-0.038(-0.049, -0.028)** | **<0.001** |  | **-0.032(-0.044, -0.021)** | **<0.001** |

***** adjusting for gender, age, nation, type of medical insurance, presence of underlying health condition, school bullying, frequency of exposure to second-hand smoke, number of friends, academic performance, evaluation of dietary status, exercise time per week, extracurricular reading time per day, average age of parents, parental education level, relationship between children and their parents, total income per year, parents working in other cities

** adoption by self-reporting

**Table S4. Association between characteristics of myopia and VAS of children in the study**

| **Characteristics of myopia** |  | **Unadjusted model** | |  | **Adjusted model*** | |
| --- | --- | --- | --- | --- | --- | --- |
|  |  | **β (95%CI)** | ***P*-value** |  | **β (95%CI)** | ***P*-value** |
| **Refraction status** |  |  |  |  |  |  |
| Emmetropia |  | ref | ref |  | ref | ref |
| Myopia |  | **-2.093(-3.057,** **-1.128)** | **<0.001** |  | -0.832(-1.934, 0.271) | 0.139 |
| **SE, diopters** |  | **0.666(0.433, 0.899)** | **<0.001** |  | **0.384(0.105, 0.663)** | **0.007** |
| **ETDRS** |  | **0.119(0.076, 0.163)** | **<0.001** |  | **0.091(0.049, 0.133)** | **<0.001** |
| **Eye health self-evaluation** |  |  |  |  |  |  |
| General |  | ref | ref |  | ref | ref |
| Bad |  | **-3.302(-5.230, -1.374)** | **0.001** |  | **-2.246(-4.144, -0.348)** | **0.020** |
| Good |  | **3.012(1.976, 4.048)** | **<0.001** |  | **1.967(0.910, 3.023)** | **<0.001** |
| **Visual acuity change in previous year**** |  |  |  |  |  |  |
| No change |  | ref | ref |  | ref | ref |
| Myopia onset |  | -0.927(-2.024, 0.171) | 0.098 |  | -0.334(-1.473, 0.804) | 0.565 |
| Myopia progression |  | **-5.345(-6.995,** **-3.696)** | **<0.001** |  | **-3.563(-5.330, -1.796)** | **<0.001** |

***** adjusting for gender, age, nation, type of medical insurance, presence of underlying health condition, school bullying, frequency of exposure to second-hand smoke, number of friends, academic performance, evaluation of dietary status, exercise time per week, extracurricular reading time per day, average age of parents, parental education level, relationship between children and their parents, total income per year, parents working in other cities

** adoption by self-reporting
